# Progress in Obstetrics and Gynecology1The Second Series of Harrington Lectures Delivered at the University of Buffalo, May 24, 25, 26, 1910.

**Published:** 1910-10

**Authors:** J. Clarence Webster

**Affiliations:** Chicago Ill.; Professor of Obstetrics and Gynecology in Rush Medical College, affiliated with the University of Chicago


### BUFFALO MEDICAL JOURNAL

Vol. Lxvi. OCTOBER, 1910. No. 3

### ORIGINAL COMMUNICATIONS

### Progress in Obstetrics and Gynecology[1]

By J. CLARENCE WEBSTER, M.D.
Chicago Ill.
Professor of Obstetrics and Gynecology in Rush Medical College, affiliated with the University of Chicago.

Lecture III.—Gynecology—Part II.

HISTORICAL research proves that the ancients knew more about gynecology than obstetrics. From references in the old writings to works which are no longer extant, it would appear that in some parts knowledge was much more extensive than is generally believed. If we had complete records, for example, of the Alexandrian schools in their best period, we would doubtless be very much surprised, not only at the variety of the diseases with which the masters were familiar, but at the therapeutic measures which were employed to cure them. The following brief references are interesting in this connection :

Rigidity and stenosis of the cervix were described by Hippocrates, Soranus, and later writers. Dilatation by means of wooden and metal rods, flax and sponge tents, was used to change these conditions. Hematometra, hydrometra and pyometra were well described by Hippocrates. Laceration and erosion of the cervix were recognised and treated. The author of the work on gynecology wrongly attributed to Hippocrates gives a good description of chronic metritis, as well as of septic metritis resulting from abortion.

Various authors describe all the known uterine displacements, some discussing their etiology. Aetius has given an account of their symptomatology and of methods of correcting the deviations by the knee-chest position, as well as by the sound. The etiology and symptomatology of prolapsus were very considerably understood at the beginning of the Christian era, the condition being treated by the use of astringent tampons and various forms of vaginal plugs.

1. The second series of Harrington Lectures delivered at the University of Buffalo, May 24, 25, 26, 1910.

Inversion was known, Soranus clearly pointing out that it might be caused by traction on the cord. Carcinoma was recognised in very early times, Hippocrates having pointed out its incurability. Fibroid tumor is not clearly described, but various ancient writers seem to have included this growth with other swellings under the term "mole." Calculus of the uterus was described by Hippocrates and Aetius, and can only refer to calcification of a fibromyoma.

Pelvic inflammation, suppurative and non-suppurative, and hematocele were noted by various writers. Soranus referred to inflammatory swellings of the adnexa and described a case of inguinal ovarian hernia. Ovarian cysts were probably described as abdominal dropsy and, like the latter, were often tapped.

Atresia of the cervix and vagina was known, and the operative treatment of the latter has been given by Celsus and Aetius. They also pointed out the importance of keeping the opening patent after the operation. Malignant growths and inflammatory affections of the outer genitals were described by many. Under certain conditions vulvar abscess was treated by incision.

Clitoridectomy was described by Aetius. That venereal disease existed in the times of these various writers and even earlier can scarcely be doubted, though it does not appear that gonorrhea was recognised as an important etiological factor in the production of disease in women. The Jerusalem Talmud has various references which clearly describe this disease. The Arabians recognised its importance as an etiologic factor in affections of the bladder and testicles.

As regards abdominal examination, inspection, palpation, and percussion were practised as early as the times of Hippocrates. Long before his time the Egyptians practised digital vaginal examination. Though the bimanual method never seems to have been generally employed, it is distinctly described by Aetius in the diagnosis of stone in the bladder and uterine tumor.

Rectal examination by the finger and speculum was known and dilatation of strictures by bougies practised. The lithotomy position for the investigation of gynecologic troubles was described by Archigenes (referred to by Aetius). The Hindus also employed it in rectal operations. The lateral position was used and the knee-chest posture is referred to by Aetius in the correction of retroflexion of the uterus.

An inverted position of the body (the extreme Trendelenburg of today) was practised very early in the treatment of procidentia, being described by Hippocrates. The vaginal, as well as the rectal speculum is a very ancient instrument, having been used in ancient India and Greece, the earliest forms being many-bladed. The sound was used by Hippocrates to dilate the uterus and to correct displacements. It was also used to measure the vagina. Special dilators of wood and metal were also employed. Aetius refers to Archigenes as also using sponge tents, while various sizes of flax tents were recommended by Hippocrates. Albucases in the twelfth century describes a metal dilator with expanding blades.

Catheters were used for emptying and irrigating the bladder. Among the Hindus the injection was made by means of a dried bladder of the cow or pig, a forerunner of our rubber reservoirs. Irrigation of the uterus was carried out in diseased conditions. Hippocrates used metal catheters for this purpose, the fluid flowing from a dried pig's bladder attached to the catheter. Vaginal and rectal injections, sitz-baths, vapor baths and fumigations of the uterus and vagina were widely employed in ancient times.

Hot fomentations and poultices were common forms of treatment. Dry heat was also applied by means of vessels of hot oil or water. Leeching, cupping and bleeding are described by many authors in the treatment of gynecological diseases.

The cautery was used in opening abscesses, in destroying disease and in checking hemorrhage. In incision of the breast, Galen used a cautery knife,—a razor heated in fire. Cauterization with arsenic and other substances was known. General and local massage was practised in very ancient times among many nations.

Pessaries and suppositories were used by the Egyptians and Hindus as well as by the Greeks and Romans. Vaginal wool tampons soaked in medicines were also widely employed. In checking hemorrhages cold applications, styptics and the cautery were widely used. The ligature was used in the time of Celsus. Various authors refer to torsion of vessels as a valuable agency. The Arabians undoubtedly used catgut for ligatures and sutures, Albucasis referring to the sewing of wounds with thread made of the twisted gut of animals.

Owing to the lack of free communication between various countries in ancient times, the scanty dissemination of knowledge by means of books, together with the overthrow of nations by war it is not surprising that medical knowledge was not transmitted from one generation to another, as is the case in modern times under more favorable conditions. At the time of the Italian renaissance far more had been lost or forgotten than had been retained. Indeed, at the beginning of the nineteenth century European gynecology was but a moiety of the combined knowledge of the subject as it was known to the best Greek and Arabian masters.

If we examine the textbook of Dr. Thomas Denman, the leading English practitioner in gynecology in 1805, we are amazed to find that his account refers only to enlargement of the clitoris, excrescences which form at the meatus urinarius, uterine polypus, and ovarian dropsy. Bimanual examination at this period was unknown and even abdominal palpation was very imperfectly carried out.

In Gooch's *Account of some of the most important diseases peculiar to women*, published in 1831, the greater portion refers to obstetric conditions, diseases of children, and the plague. He devotes 47 pages to uterine polypi and a few to irritable uterus. Blundell's work (1837) was of a much higher order. He described uterine displacements and inversion, carcinoma, polypi, bladder diseases, ovarian dropsy, and functional diseases. He suggested (though he did not carry out in practice) division of the Fallopian tubes at the time of Cesarean section for contracted pelves, extirpation of the ovaries for dysmenorrhea and hemorrhage following uterine inversion, Porro's modification of the Cesarean operation, and extirpation of the cancerous uterus.

In the fourth decade of the nineteenth century, ulceration of the neck of the womb was the chief condition for which women were treated, Dr. Henry Bennett attributing nineteen out of twenty cases of women's diseases to this condition. There are well founded traditions of fabulous fortunes made by fashionable practitioners, who devoted their lives to the touching of these diseased spots with various applications. Hodge, of Philadelphia, previous to 1860, was one of the strongest opponents of the prevalent "ulceration" pathology and the clinical views founded upon it. Years afterward he was supported by many workers, especially by Ruge and Veit, who showed that the so-called "ulcer of the cervix" was no ulcer at all, but merely the extension outward of the columnar epithelium of the cervical canal, displacing the squamous epithelium of the portio vaginalis.

The greatest advance in the accurate diagnosis of pelvic diseases was due to

1. Improved abdominal palpation.
2. Use of the uterine sound.
3. Bimanual examination.

Sir James Y. Simpson deserves chief credit for insisting on these methods. He reintroduced the sound and showed how it could be used with the bimanual examination. His pioneer views have been greatly modified, for the sound has been almost entirely discarded in diagnosis, but his work was instrumental in directing attention to the need of thorough local examination. In his memoir on the sound he pointed out that this had been generally disregarded, and that gynecological diagnosis was based on the following considerations:

1. The state of uterine functions and those of neighboring viscera.

2. The existence of sympathetic mammary or spinal pains.
3. The condition of patient's health.

If Simpson had devoted as much attention to the bimanual examination as he did to the sound, the history of gynecological progress in the last fifty years would have been considerably different from what it has been, and the clinical discovery of diseased tubes and ovaries would not have been so long delayed. As it was, the introduction of the sound led to a most disproportionate consideration of uterine flexions and versions as the chief causes of women's pains and troubles, much ingenuity being exercised in devising pessaries to correct these distortions and deformities. This was the happy era when suffering women found relief in the office administrations of the zealous practitioner, and must have been in Clifford Albutt's mind when he referred to the patient "entangled in the web of the gynecologist, who finds her uterus, like her nose, a little on one side; or again, like that organ, running a little; or as flabby as her biceps, so that the unhappy viscus is impaled upon a stem, or perched upon a prop, or is painted with carbolic acid every week in the year, when the gynecologist is grouse-shooting, or salmon-catching, or leading the fashion in the upper Engadine."

In the midst of this era, gynecology received a new impulse as a result of a renaissance in repair surgery, due mainly to the efforts of Marion Sims and Bozeman of America, as well as to the establishment of ovariotomy as a feasible operation. The pioneer work of McDowell in America had never impressed the public and had been almost forgotten. Charles Clay's work in England in the early forties, splendid though it was for the time, attracted little attention. It remained for Spencer Wells to show the profession that ovarian tumors could be successfully dealt with by surgery. In America, Atlee was to a great extent responsible for the popularising of the operation.

Thomas Keith, of Edinburgh, soon afterward entered the field and taught the profession that uterine fibroids could be successfully removed by abdominal surgery. This distinguished surgeon attained results, even before the Listerian era, which were remarkable. Indeed, for several years, he stood alone as the one successful operator in cases of fibromyoma uteri. This was mainly due to his technic. He was careful as regards cleanliness, his standard being in effect an aseptic technic, and he employed drainage with great freedom.

The next great advance was made in the treatment of diseases of the uterine appendages. Tubal diseases had been neglected though recognised and somewhat described by Cruveilhier, Boivin and Duges, Hooper, Bernutz, Goupil and others; but no rational treatment was carried out until 1872, when, within a period of six months, Battey of Georgia, Lawson Tait of England, and Hegar of Germany did their first operations. The former removed the ovaries from a young woman subject to marked neurotic symptoms associated with menstruation. Tait's operation was the removal of the appendages in a case of bleeding fibroid, while Hegar's procedure was to cure "ovarian neuralgia." To these pioneers suffering women have owed an enormous debt of gratitude, in spite of the fact that the operation has been vastly abused and often misapplied.

As Lister's great discovery became gradually known, and diseased conditions in the pelvis and abdomen were attacked with more boldness, the era of modern gynecology really began. Clinical observations were accompanied by laboratory research, and the study of women's diseases was at last established on a scientific basis. General surgery is greatly indebted to the work of gynecological operators, for their brilliant results blazed the path for the exploration of the entire abdomen and other body cavities.

On reviewing the history of gynecology during the last hundred years, it would not be amiss to describe it as a succession of "fads and fancies," as late, at least, as the present scientific era. Throughout, there has been an amazing exercise of the speculative habit, bad logic, imperfect clinical examination, and lack of study of *post-mortem* material. When Bernutz and Goupil published their splendid pathological work relating to the pelvis, little attention was paid to it.

What now about the present? Are we entirely free from the faults of mind of our predecessors? Is the logical and scientific habit so universally prevalent that our work represents the highest possible achievement? In the early days of my professional work, owing to the marked trend in gynecological practice, a narrow and debased specialism had been gradually evolved, resulting in the establishment of a school whose motto was Michelet's dogma, *Le bassin c'est la femme,* and whose remedial measures were limited to different forms of mechanical procedure, from repairing the cervix to removing the ovaries. The opprobrious denunciation heaped upon this school has not been unmerited. Too strong a protest cannot be urged against the centralisation of attention on local pelvic conditions, regardless of wider physical and psychical relationships.

Pascal has a chapter in his famous book, entitled "Man's Disproportion." The term might justly be applied to the mechanical school of gynecologists, who have done so much harm, by their failure to give to the various symptoms related to the pelvis their proper proportional values.

It is in the treatment of neuroses in women that the modern gynecologist has shown himself at his worst. In the correction of bent wombs, and repairing of slight tears he has been little better than Bennett of old, who believed that touching a supposed ulcerated cervix was a cure for all women's troubles. We cannot value too highly the important work of men like Playfair, Clifford Albutt, Weir Mitchell, Beard and others in analysing the neuroses of women and in establishing the treatment of neurasthenia on a rational basis.

That neuroses should be so common in women is not to be wondered at. Though Michelet's statement that "woman's life is a history of disease" is not quite accurate, it must be admitted that it is prone to physiologic unrest and pathologic changes unknown to man. When we remember the marked disturbances which accentuate the advent and departure of the reproductive era of her life,—the profound changes taking place during ovulation, menstruation, pregnancy, labor and lactation; the subtle and complex activities of physical life in its various diastaltic functions,—it is not remarkable that neuroses should manifest themselves particularly in relation to her reproductive mechanism.

That they are increasing, *pari passu,* with the advance in our highest civilisation cannot be denied,—among the poor, the inducing factors being overwork, overworry, ill-regulated and poor nutrition and bad hygienic surroundings; among the well to do, educational overstrain, overindulgence, the stress of artificial life and emotional excitement. A condition is developed in which the whole nervous system is below par, the manifestations being irregular or excessive activity, abnormal sensitiveness, mental unrest and anxiety. Though there is a deficiency of nerve force, there is an increase in energising, due to a weakness in inhibitory power and to the too ready response of the nervous system to stimuli.

In the worse stages of the condition the increasing excitability may change to a state in which there is an actual obtunding of nerve sensibility. Hyperesthesia and motor weakness, then, are the chief features of neurasthenia. Abnormal sensations and pains may be felt in different parts of the body. Cramps and twitchings may develop. The pupils may be unequal in size; the tendon reflexes are often exaggerated; there is often, too, a feeling of languor and of unfitness for work. The appetite fails; pains in the stomach and bowels may develop, along with indigestion, constipation, and the like. Emaciation is common, though in some cases, fat is increased, the patient at the same time, however, appearing pale and unhealthy. The urine is often of low specific gravity, may contain deficient urea, and often abundant phosphates. Sleeplessness frequently develops. The woman may become very anxious and be subject to various fears; numerous other abnormal emotional and mental symptoms may gradually become established and in extreme cases, some form of insanity may arise. Neurasthenia may lead to hysteria, and may have symptoms which are found in hysteria, but it is important to keep in mind the great differences between the conditions.

As Albutt puts it, neurasthenia is the state in which there is "defect of indurance," hysteria that in which there is "defect of the higher gifts and dominion of mind," the higher nervous centers being at fault. In the hysteric there is an abnormal degree of response to suggestion, a partial or complete loss of inhibition in regard to voluntary actions, and a disturbance of the centers regulating the automatic movements. The powers of the higher centers may greatly alter in regard to the initiation of movements, sensory perception may be partially or entirely wanting.

Grateful as we must be to the physicians and neurologists who have insisted upon the multiformity of the manifestations of the neurasthenic state, we must not be sparing in our criticism of them when they have tried to underrate the importance of real pathologic changes in the genital organs, in inducing many of these manifestations. Valuable as are the therapeutic measures employed by the physicians to counteract the disturbances of neurasthenia—namely, change of scene and occupation, freedom from overwork, worry, emotional excitement, improved nutrition, massage, together with mental suggestion, encouragement, insistence on self-control, relaxation, and avoidance of introspection, they will often be useless in inducing permanent cure if disturbing pathologic conditions in the pelvis and abdomen are neglected.

Some very prominent specialists in the treatment of neurasthenia, by erring in this direction have made blunders as serious as those made by the much-abused gynecologist whose range of vision has been restricted to the pelvic organs. Might I state that within a year I have seen two cases each treated for neurasthenia by prominent neurologists for a period of six or eight weeks, in which no physical examination had been made at all. In one, a married woman, I found bilateral chronic tubo-ovarian abscesses, not producing much pelvic pain and without a history of acute onset. The removal of these masses effected what all the ministrations of the neurologist had been unable to accomplish.

The other case was more instructive. It was that of a girl of twenty-one, engaged to be married. For years she had been under the care of a physician notorious for his opposition to surgery. For several years she had many manifestations of neurasthenia, with irregular and scanty menstruation, marked constipation and irritability of the bladder. She had been abundantly dosed with iron and other tonics, and had been sent to health resorts by the seaside and in the mountains, without very much change. Finally, as marriage was impending, the doctor sent her to a distinguished physician, prominent for his skill in dealing with the neuropathics of the fair sex. For eight weeks she was under the influence of his impressive personality. He cajolled her, bullied her, stuffed her with milk and eggs, but, as the patient herself expressed it, he never laid hands on her. In other words, no physical examination of any kind except a blood analysis had been made. When she returned to Chicago, a chastened though not a healthier woman, a friend induced her to see a plain ordinary gynecologist, who found the following condition: atresia of one-half of a bicornuate uterus with distension of the horn and corresponding tube forming a mass which filled a large portion of the pelvic cavity, interfering with the functions of the rectum and bladder to a very considerable extent. Under the circumstances, even the family doctor agreed that surgical measures should be tried.

From a pretty extensive experience with women, I am convinced that careful investigation will considerably reduce the percentage of cases of neurasthenia in which the causes are intangible or undiscoverable, with a consequent improvement in the results of a logical and rational therapy. But this great desideratum will not be attained if gynecologists restrict their attention to the pelvis, and if physicians and neurologists forget that various forms of trauma and diseases affecting the pelvis and abdomen, may be the starting point of a variety of symptoms, local or distant, affecting sensory, motor, circulatory, visceral or even mental functions.

In the last twenty-five years the investigation of the entire abdominal cavity has revealed a variety of diseased conditions which had been previously to a great extent unrecognised, and which are now known to be responsible for symptoms which have far too often been attributed to neurasthenia. I refer to diseases of the kidneys, stomach, appendix, and gall-bladder. Of great importance are visceral displacements, termed ptoses.

Glenard first pointed out in 1885 the importance of downward dragging of the large intestine in the production of neurasthenic disturbances and, though many of his views have been discredited, his work was responsible for directing attention to this very important subject. Though the nature of the normal relationships of the abdominal and pelvic viscera and the method by which they are altered are not yet thoroughly understood, great progress has been made in the elucidation of these problems.

Important as the specialty of gynecology is, it can no longer be limited as it has been in the past. A successful worker in this branch must give his attention to the entire abdomen and he cannot afford to be ignorant of obstetrics. Owing to the limited facilities afforded in most medical schools for the study of the special diseases of women, those who would attain to excellence in work must necessarily carry on prolonged post-graduate study as assistants to able teachers. As surgery plays such an important part in the treatment of the specialty, a strenuous apprenticeship in the operating room, whereby thorough drill in aseptic technic can alone be obtained is all important.

The term "aseptic surgery" has come to be regarded in the minds of many as a kind of mascot, which is supposed to permit operative procedures to be carried out on the human body under any circumstances, without let or hindrance. The fascination of exhibiting manipulative dexterity too often destroys the sense of proportion and makes the operator oblivious to other considerations. It is, therefore, easy to understand why there may often be found a tendency to neglect conditions which might be contraindications to surgical procedures, or might lead to a modification of them. In pre-Listerian days, there was some excuse for the concentration of the surgeon's attention on the mechanics of surgery; in the present era, much more is required of him.

The careless use of anesthetics is responsible for an unnecessary number of deaths both immediate and indirect. It is very easy to fall into a routine administration, neglecting to give careful individual consideration to the proper selection and use of an anesthetic, or to exercise supervision over the anesthetiser. In many serious cases, a fatal issue may result from the use of chloroform or ether, which might be avoided by the use of nitrous oxide or oxygen, for example, or local anesthesia. The administration of anesthetics is too often left to those who have had no special instruction in the art, a prevailing opinion being that no special fitness or preparation is required. Too strong a protest cannot be raised against such practice. The greatest degree of safety and efficiency is obtained only when the anesthetiser combines thorough knowledge with practical experience.

But it is the actual work in the operating room to which I desire to call special attention. In pre-Listerian days, the surgeon concerned himself only with the mechancial details of his operation. Knowing nothing of the causes of wound infection, though full of dread with regard to the occurrence, his chief object was to perform his work with speed and dexterity. Under modern conditions, the manipulations of the operator are but part of a series of activities, each of which is of the greatest importance in determining the outcome of surgical work.

The sterilisation of instruments and dressings, the preparation of solutions and suture material, the cleansing of the skin and other details of technic require constant supervision and scrutiny. Too often is this necessary oversight lacking on the part of operators, with the result that faults and imperfections creep into the system and remain there.

Modern technic is simple in theory, but its practical details are elaborate and cannot be disregarded by the successful operator. He should insist upon the methodic training of the hands and eyes of his assistants and nurses, so that the habit of working with precision may be required. Perfection in work, whether of the individual or of the combined staff, is usually automatic and without conscious effort. Yet this standard is reached only after a long period of training, the minutest details being often repeated, in the early stages, with conscious effort, guided by an active intelligence. It is only gradually that the habit is acquired of acting without special attention. Unless this method of education be pursued, bad habits are certain to be formed so that the operator or his staff, in crises or emergencies, or even in ordinary circumstances, may easily deviate from the proper line of action, and commit more or less serious errors in technic. Yet, in the surgical world comparatively few have subjected themselves to this rigorous training.

A knowledge of the most important facts in bacteriology is essential to the greatest success, and it is advisable that this information should be gained by practical work in a bacteriological laboratory. The censorship of the latter should always be welcomed by the operating room, for by this alone can the failures to obtain perfect asepsis be determined. The limitation of our knowledge and other considerations may place absolute perfection in surgical work beyond our reach, but it is certain that the irreducible minimum of fatality in surgical work may be brought considerably nearer to the vanishing point, if operators give as much attention to bacteriology, physiology, self-discipline and team training as they do to the mechanics of their craft.

The rapid increase in the numbers of those who undertake all kinds of surgical work without proper preparation or control, is not in the best interests of the public. I drew attention to this subject in an editorial in the Journal of the American Medical

Association a few years ago, and it does not seem ill-fitting on this occasion to repeat my statements and to call the attention of the profession to an undoubted growing evil.

A marked characteristic of the present generation is the rapid multiplication of hospitals both in urban and rural communities. That such an extension of these institutions has been a boon must be universally admitted. But it cannot be denied that the benefits are in many instances accompanied with a serious evil, the practice of surgery by the irresponsible and untrained. In the leading European countries surgical practice is almost entirely in the hands of those who have been specially trained under competent masters, the period of apprenticeship amounting to five or more years in the great majority of instances.

A medical man, whether old or young, would find it practically impossible to obtain a surgical practice without such a preliminary training. He would be frowned upon by the rest of the profession and by the public. In Great Britain additional requirements are necessary, for in most hospitals staff appointments are given only to practitioners who in addition to having had special training, have obtained extra qualifications from either the College of Surgeons, or the College of Physicians. These qualifications are obtained only through difficult examinations, and they are a guarantee that candidates have made a thorough post-graduate study of anatomy, pathology, bacteriology as well as of practical medicine and surgery.

Maurice Richardson referring to conditions in America, without specifying any special community or man, says: "there is no doubt whatever that surgery is being practised by those who are incompetent to practise it—by those whose education is imperfect, who lack natural aptitude, whose invironment is such that they never can gain that personal experience which alone will really fit them for what surgery means today. They are unable to make correct deductions from histories; to predict probable events; to perform operations skilfully; or to manage aftertreatment. Surgical operations should be performed only by those who are educated for that special purpose."

If these remarks be true, a heavy responsibility rests upon the best elements in the medical profession, upon our educational institutions and upon State Boards of Health. Unless medical standards are changed from within the profession they are likely to be rudely changed by forces acting from without. Irresponsible, amateurish surgery means increased mortality and morbidity. The sufferers are the public. It will not be surprising if they institute reforms and demand supervision of hospitals and of the credentials of those responsible for the surgical work. There is no reason why the records of operations should not be open to supervision.

All conscientious workers, who have spent years in training and improving themselves for the responsible work of surgery, and who best know the dangers and difficulties of its practice, must stand together and support such leaders as Maurice Richardson in their demand for a better scientific and practical education, as well as for a keener sense of responsibility on the part of those who meditate a surgical career. Opportunities should be afforded by leading schools and hospitals for giving the proper training. Graduates should be encouraged more and more to adopt the European system, of spending several years in severe and unremunerative apprenticeship, and their reward should be responsible positions which afford them the opportunity of developing towards a ripe surgical maturity, in the full confidence of their professional brethren and the public.

